# Exosomal Non-Coding RNAs: Regulatory and Therapeutic Target of Hepatocellular Carcinoma

**DOI:** 10.3389/fonc.2021.653846

**Published:** 2021-03-26

**Authors:** Haoming Xia, Ziyue Huang, Shuqiang Liu, Xudong Zhao, Risheng He, Zhongrui Wang, Wenguang Shi, Wangming Chen, Zhizhou Li, Liang Yu, Peng Huang, Pengcheng Kang, Zhilei Su, Yi Xu, Judy Wai Ping Yam, Yunfu Cui

**Affiliations:** ^1^ Department of Hepatopancreatobiliary Surgery, Second Affiliated Hospital of Harbin Medical University, Harbin, China; ^2^ The Key Laboratory of Myocardial Ischemia, Harbin Medical University, Ministry of Education, Harbin, China; ^3^ Department of Pathology, Li Ka Shing Faculty of Medicine, The University of Hong Kong, Hong Kong, Hong Kong

**Keywords:** exosomes, non-coding RNAs, hepatocellular carcinoma, tumor microenvironment, early diagnosis, precise therapy

## Abstract

Exosomes are small extracellular vesicles secreted by most somatic cells, which can carry a variety of biologically active substances to participate in intercellular communication and regulate the pathophysiological process of recipient cells. Recent studies have confirmed that non-coding RNAs (ncRNAs) carried by tumor cell/non-tumor cell-derived exosomes have the function of regulating the cancerous derivation of target cells and remodeling the tumor microenvironment (TME). In addition, due to the unique low immunogenicity and high stability, exosomes can be used as natural vehicles for the delivery of therapeutic ncRNAs *in vivo*. This article aims to review the potential regulatory mechanism and the therapeutic value of exosomal ncRNAs in hepatocellular carcinoma (HCC), in order to provide promising targets for early diagnosis and precise therapy of HCC.

## Introduction

Hepatocellular carcinoma (HCC) is the third most common malignant tumor worldwide, accounting for 85–90% of primary liver cancer (1–3). Chronic liver inflammation and fibrosis mediated by viral hepatitis, alcohol and non-alcoholic fatty liver disease (NAFLD) have become the initiators of HCC (4, 5). Due to the lack of obvious early clinical symptoms and biomarkers, most patients have reached the advanced stage at the time of diagnosis, losing the opportunity of curative treatment (resection or transplantation) as a result of multiple intrahepatic and distant metastases. Palliative care has become the only option for advanced-stage patients (6, 7). The 5-year survival rate of less than 20%, the high resistance of palliative care and the frequent relapse make early diagnosis an inevitable requirement for prolonging the survival time of HCC patients (8). Relying on capturing humoral cells and nucleic acids including circulating tumor cells (CTC), circulating tumor DNA (ctDNA) and exosomes, liquid biopsy, an emerging minimally invasive technique, is helpful for the early diagnosis and monitoring of disease progression or treatment response of cancers (9). However, the low peripheral blood abundance of CTC and the short half-life of ctDNA make exosomes a better choice.

Extracellular vesicles (EVs) are submicron particles bounded by phospholipid bilayers with non-functional nuclei, which are naturally released by a variety of cells and have no replication function. “EV” is a term proposed by International Society for Extracellular Vesicles (ISEV) (10), covering exosomes, microparticles, apoptotic blebs and other EV subsets. Exosomes can mediate cross-linking between tumor cells/non-tumor cells and recipient cells by carrying a variety of biologically active substances (protein, RNA, cholesterol, lipids, etc.), as well as modifying the recipient cells phenotype and reshaping the TME through cargo transfer (11, 12). For example, exosomes derived by breast cancer cells can regulate multiple molecules and signaling pathways in receptor cells by carrying miR-155, miR-21, miR-1246 and other cargo, including TGF-β/Smad signaling, Mef2c-β-Catenin, etc., which mediate tumor cell proliferation, metastasis and radio/chemoresistance (13). Irradiation can induce the high secretion of irradiated cells and the pathological changes of its internal cargo, participating in the formation of the bystander effect of unirradiated cells, which is manifested by increasing the cell growth rate and inducing biological behaviors such as radioresistance by repairing damaged DNA (14). Annexin II, Heparanase, TGFβ, miR-126a and other angiogenic cargo can activate angiogenesis through targeted transport between tumor cells and endothelial cells (15). Moreover, exosomes are highly secreted in cancer patients. Benefit by their wide distribution in multiple body fluids (blood, urine, saliva, etc.) and the differential expression of cargo during the development of cancer, the isolation and acquisition of exosomes and the expression analysis of its cargo have become feasible, which are conducive to the early diagnosis, treatment and prognosis evaluation of cancers (16–18). For this reason, the high stability and low immunogenicity of exosomes make them a natural vehicle for targeted anti-tumor drug/nucleic acid delivery *in vivo*.

Non-coding RNAs (ncRNAs) are a kind of RNA subgroups without protein-coding ability or only have short peptide coding ability, including miRNAs, long ncRNAs (lncRNAs), circular RNAs (circRNAs), tRNA-derived small RNAs (tsRNAs), PIWI-interacting RNAs (piRNAs) and pseudogenes, etc. NcRNAs are widely and selectively enriched in exosomes, regulating gene expression at the transcriptional, post-transcriptional and epigenetic levels (19). Numerous studies have confirmed that the potential regulation of tumor metastasis, angiogenesis, immune escape, metabolic reprogramming and drug resistance make exosomal ncRNAs become endogenous tumor regulators in the body (20), and their dysregulation directly participates in the onset and progression of carcinogenesis (21, 22). This article aims to outline the potential regulatory mechanisms of exosomal ncRNAs in HCC, with a view towards providing options for the clinical application of HCC-related biomarkers and therapeutic targets.

## Overview of Exosome

### Exosome Biogenesis

**Figure 1:**
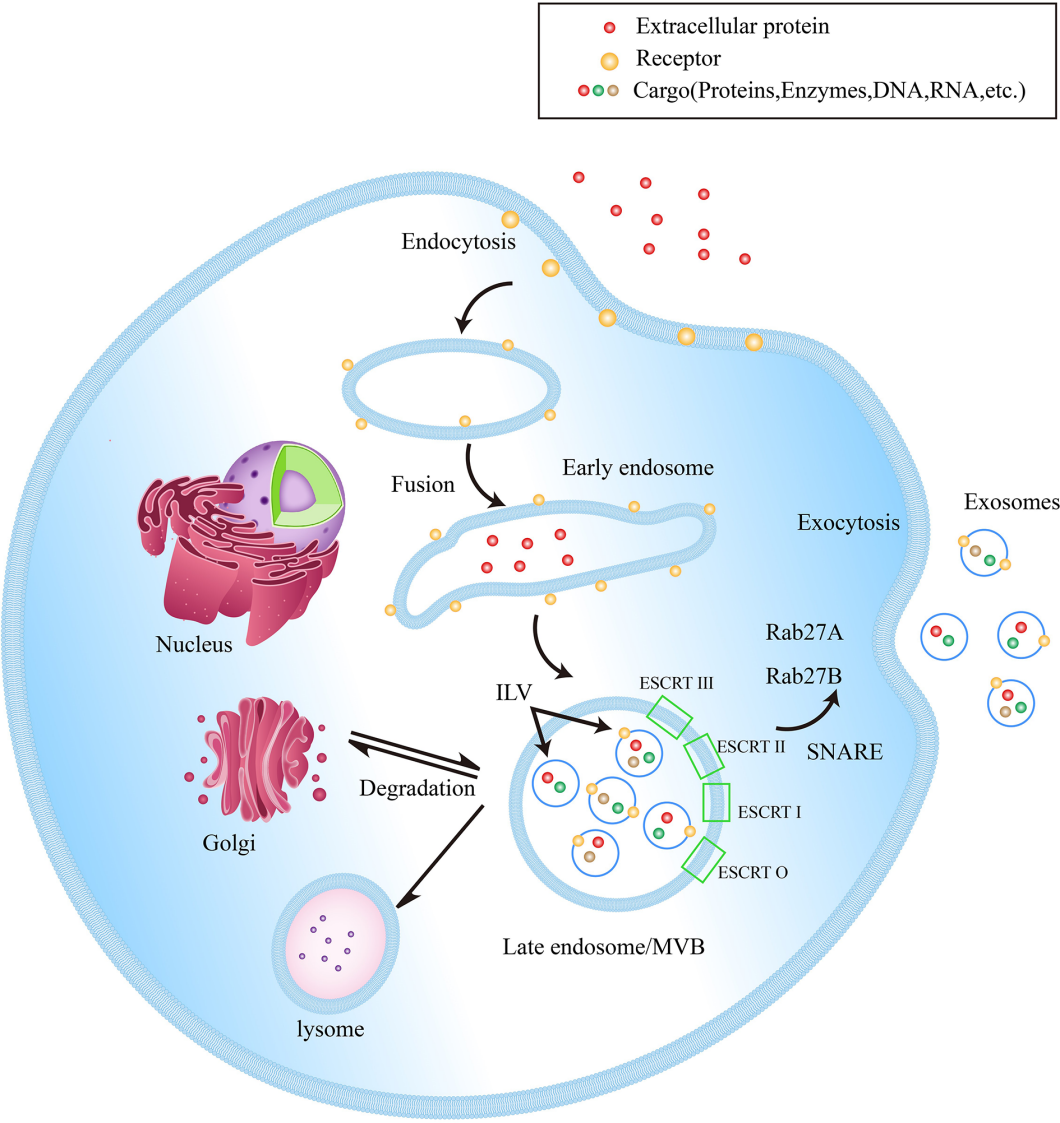
Exosome biogenesis within endosomal system. Early endosomes are formed by the fusion of endocytic vesicles, further transformed into late endosomes/MVBs through inward budding of the membrane (packing the cargo into ILVs). ILVs protein sorting has two methods, ESCRT-dependent and ESCRT-independent. ESCRT-0, ESCRT-I, ESCRT-II and ESCRT-III are the four components of the ESCRT. They generalize the substrate on the inwardly budding endosomal membrane. Subsequently, a part of MVBs is fused with lysosomes to be degraded, while another part of MVBs can be fused with the plasma membrane to release ILVs into the extracellular space, now called exosomes. In addition, Rab27A and Rab27B are important mediators leading MVBs to the periphery of cells, and the SNARE complex helps MVBs fuse with the plasma membrane.

EVs, with lipid bilayer-enclosed membranous structures, are induced by cells to secrete under physiological conditions or specific biological instructions, which can be classified into different heterogeneous populations according to different sizes and subcellular origins (10). Since the specific biomarkers of different EVs have not been clearly defined yet, exosomes specifically refer to small EVs with a size of 40-160 nm (23). The process of exosome biogenesis includes three key steps: 1. The exosomes are initially formed as intraluminal vesicles (ILVs) from late endosomal membrane invagination. 2. ILVs form multivesicular bodies (MVBs) through endosome sorting complexes in an ESCRT-dependent or independent pathway. 3. MVB fuses with plasma membrane to release exosomes (24). **Figure 1** shows the biogenesis of exosomes

### Exosomes Mediate Intercellular Communication

Since it was first discovered in mature reticulocytes in the 1980s (25), the extensive participation of exosomes in different pathophysiological processes such as viral infection, immune response, and tumor metastasis has been successively confirmed (26). Relying on the specific carrying of various biologically active substances (including protein, RNA, cholesterol, lipids, etc.), exosomes establish a contact-free substance exchange between cells in autocrine, endocrine, proximal, paracrine and remote communication (27). For example, myelin oligodendrocytes count on exosomal secretion to promote axon transport and long axon maintenance in adjacent neurons (28). Adipose stem cell-derived exosomes inhibit ischemia-reperfusion injury of cardiomyocytes by targeting miR-221/miR-222/PUMA/ETS proto-oncogene 1 (ETS-1) pathway (29). Exosomal Hic-5 regulates the proliferation and apoptosis of osteosarcoma *via* Wnt/β-catenin signal pathway (30).

Exosomes transmit signals *via* ligands/adhesion molecules expressed on the membrane surface or the cargo carried inside (31), establishing two crosslinking pathways with the recipient cells, either by membranous interactions or by payload release following cell uptake of exosomes (32). The exosome-mediated communication between donor cells and recipient cells has the following characteristics: 1. MVB-derived exosomal membranes contain ligands that can simultaneously bind to multiple cell surface receptors, therefore providing an information network in a way that highly mimics the direct contact between cells and cells (33). 2. The internalization of exosomes into recipient cells results in “novel” cell membrane loading part of the donor cell surface molecules, thereby changing the original adhesion or recruitment characteristics (34). 3. Exosomes provide an ideal medium for delivering genes or proteins to reprogram target cells (35). Additionally, as shown in **Table 1**, numerous studies have revealed the regulatory role of plentiful ncRNAs in HCC.

**Table 1:** Exosomal ncRNAs in HCC.

ncRNAs	Functions	Targets/Substrates	References
**lncRNAs**			
SENP3-EIF4A1	suppresses the proliferation and migration of HCC cells *via* SENP3-EIF4A1/miR-9-5p/ZFP36	miR-9-5p/ZFP36	(36)
FAM72D-3	promotes the proliferation and inhibits the apoptosis of HCC cells	EPC1-4	(37)
HULC	promotes secretion of exosomes from HCC cells, inducing cell invasion by targeting miR-372-3p/rab11a	miR-372-3p/rab11a	(38)
Linc-ROR	promotes the proliferation and prevents the apoptosis in condition of lacking nutrition	OCT4, NANOG, SOX2, P53 and CD133	(39)
FAL1	promotes cell growth and proliferation, cell migration and invasion in HCC cells.	miR-1236	(40)
**circRNAs**			
circTMEM45A	acts as the sponge of miR-665 to promote proliferation of HCC cells	miR-665	(41)
circZNF652	promotes cell proliferation, migration, invasion and glycolysis *via* miR-29a-3p/GUCD1 Axis	miR-29a-3p/GUCD1	(42)
circCdr1as	promotes proliferative and migratory abilities *via* targeting miR-1270/AFP	miR-1270/AFP	(43)
circPTGR1	increases the numbers of migrated cells, higher levels of apoptosis, and cell arrest at the S phase in the HCC cells	miR449a/MET	(44)
**miRNAs**			
miR-155	promotes the proliferation of HCC cells through miR-155/PTEN axis.	miR-155/PTEN	(45)
miR-296	attenuates the lymphatic vessel formation ability of HDLEC cells by upregulating EAG1 and stimulating VEGF signaling.	EAG1/VEGF signaling	(46)
miR-145	represses HCC cell proliferation, invasion, and migration, partially through downregulation of GOLM1.	GOLM1	(47)
let-7i-5p	inhibits TSP1/CD47-mediated Anti-tumorigenesis and Phagocytosis of HCC	TSP1/CD47	(48)
miRNA-21	converts HSCs to CAFs *via* down-regulating PTEN and activates PDK1/AKT signaling pathway to promote angiogenesis	PTEN, PDK1/AKT signaling pathway	(49)
miR-125	suppresses HCC cell proliferation, stem cell properties and migration *via* CD90	CD90	(50)
miR-93	promotes the stimulation in cell proliferation and invasion by targeting CDKN1A, TP53INP1 and TIMP2	CDKN1A, TP53INP1 and TIMP2	(51)
miR-25-5p	promotes tumor self-seeding in HCC to enhance cell motility by upregulating LRRC7	LRRC7	(52)
miR-103	attenuates the endothelial junction integrity by directly inhibiting the expression of VE-Cadherin, p120-catenin and ZO-1	VE-Cadherin, p120-catenin and ZO-1	(53)
miR-1247-3p	suppresses B4GALT3 expression to promote β1-integrin–NF-κB signaling in fibroblasts activation.	β1-integrin–NF-κB signaling/B4GALT3	(54)

### Exosomal NcRNAs Regulate Hepatocarcinogenesis

The discovery of non-coding RNA has drawn a new blueprint for the research of genome function: The nucleus has extensive transcription capacity (even introns and intergenic sites (55), resulting in the production of short and long non-coding RNA with complex regulatory effects (56). As the most abundant biologically active substance in exosomes, ncRNAs play a pivotal role in intercellular communication. Growing evidences in recent years have revealed the regulatory effects of ncRNAs on the tumorigenesis and development of HCC: Exosomal circTMEM45A improves the proliferation and vascular invasion capacity of HCC cells through circTMEM45A/miR-665/IGF2 axis (41). Exosome-derived circ-0051443 suppresses the proliferation of HCC cells by upregulating the expression of BCL2 antagonist/killer 1 (BAK1), promoting apoptosis and G0/G1 cell cycle arrest (57). Cancer cells induce the ectopic expression of exosomal miR-1273f under hypoxia. The miR-1273f/LIM homeobox 6 (LHX6)/Wnt/β-catenin signaling pathway promotes the proliferation, movement, invasion and EMT of HCC cells even under normal oxygen conditions (58).

### Exosome Therapeutic Applications

With the in-depth study of exosomal ncRNAs on the regulatory mechanisms of various cancers, gene-targeted therapies for related pathways have improved the prospective options for cancer cure. Since the cell membrane sets an insurmountable barrier to negatively charged nucleic acids, and the genetic material is difficult to transport freely in the body due to its own fragility, exosomes, as natural carriers for intercellular cross-linking, have been pushed to the forefront of drug delivery system. The natural ability to cross biological barriers (such as the blood-brain barrier), the transport capacity of areas without blood supply (such as dense cartilage matrix), the long residence time of target tissues, and the low clearance rate associated with high biocompatibility make exosomes become ideal carriers for gene targeted-therapy. In addition, exosomes have genetic engineering plasticity. The exogenous modification of their surface proteins can be used for many purposes. For example, the addition of tissue targeting peptides on the surface of exosomes can mediate selective targeting of cargo and reduce systemic pharmacological toxicity (59).

## The Effects of Exosomal ncRNAs On Hepatitis

About 12% of cancer cases worldwide are induced by chronic infection of hepatitis virus pathogens (hepatitis B, hepatitis C, and hepatitis D) (60), of which HBV (54% of all HCCs) and HCV (31% of all HCCs) are clearly defined as risk factors for HCC (61). Persistent hepatitis and chronic viral infection cause both immune system and viral protein-mediated oxidative stress damage, directly participating in the tumorigenesis and development of HCC.

HBV, as the only DNA virus that induces genome instability through viral DNA integration, leads to the production of fusion gene products and changes in the expression of oncogenes or tumor suppressors. Ostm1 recruits RNA exosomal complexes by binding to HBV RNA to induce degradation of HBV RNA, resulting in HBV replication limit. Targeted excision of the RNA exosomal component Xonuclease 3 (Exosc3) can eliminate Ostm1-mediated HBV replication inhibition. C-terminal domain provides a targeted site for HBV-related treatments as the site of RNA exosomes (62). HBV X protein (HBx), a multifunctional viral regulator, participates in the regulation of the virus life cycle and the progress of HBV-related HCC. Deng et al. (63) confirm that peroxiredoxin 1 (Prdx1) as a cellular hydrogen peroxide scavenger can bind to HBx Cys and mediate the inhibition of HBx expression by interacting with the RNA exosomal complex component Exosc5, thereby inducing HBV RNA degradation. In another study, activating transcription factor 3 (ATF3) activates the transcriptional activity by interacting with the CRE motif (+164 to +171) in the Ski2 promoter. IL-1b acts as a positive regulator of ATF3 expression, mediating the upregulation of endogenous Ski2 mRNA and protein expression. Subsequently, Ski2/RNA exosome complex is formed to reduce the stability of HBx mRNA and induce the degradation of the transcript (64).

Myeloid-derived suppressor cells (MDSCs) maintain immune homeostasis by limiting excessive inflammatory response. Chronic HCV infection induces the accumulation of MDSCs in the peripheral blood. HCV-related exosomes mediate the upregulation of lncRNA RUNXOR and its downstream target RUNX family transcription factor 1 (RUNX1) in MDSCs, thereby promoting the differentiation and immunosuppression of MDSCs by activating the immunosuppressive molecule signal transducer and activator of transcription 3 (STAT3), negatively regulating the expression of miR-124 (65). Similar to the above research results, Thakuri et al. (66) have stated that HCV exosomes upregulate lncRNA HOTAIRM1 and its target HOXA1 in MDSCs to achieve the activation of the expression of immunosuppressive molecules (Arg1, iNOS, STAT3 and ROS), and also mediate MDSCs-induced immune suppression by downregulating miR-124. In another study, HCV-related exosomes transfer immunomodulatory viral RNA from infected cells to adjacent immune cells, driving peripheral blood mononuclear cells (PBMCs) to differentiate into MDSCs. HCV-related exosomes further mediate the upregulation of peripheral blood TFR/TFH cell ratio and induce IL-10 production by inhibiting miR-124 in MDSCs, thereby antagonizing the differentiation and function of T cells during viral infection (67).

## Exosomal ncRNAs in HCC Progression

### Angiogenesis

The newly emerging sprouting microvessels or resident blood vessels next to the malignant tumor are important participants in the proliferation and metastasis of HCC cells (68). The interaction between endothelial cells and tumor microenvironment promotes the formation of new blood vessels through genetic or epigenetic mechanisms (69). Different from resting blood vessels (70), the rapid proliferation potential, high permeability and chaotic structure of new blood vessels (71) provide a convenient channel for the distant metastasis of tumor cells. Angiogenesis is also regarded as one of the hallmarks of solid (72, 73) and hematological malignancies (74), involved in tumorigenesis and development. Since Dr. Judah Folkman discovered the tumor-promoting effect of angiogenesis, targeted therapy for anti-angiogenesis has become a research hotspot for many scientists (75). However, different from the preclinical positive results obtained in animal models, the clinical application of anti-angiogenesis therapy has not achieved the desired effect yet (76). The potential drug resistance mechanism induced by vascular heterogeneity and tumor microenvironment has become the biggest shackle in this field (77, 78).

The core transcription regulator YAP1 of The Hippo pathway is highly correlated with the expression of tumor angiogenesis factor vascular endothelial growth factor A (VEGFA), and it has been confirmed to be recruited around HCC blood vessels, participating in mediating angiogenesis. The YAP1 inhibitor verteporfin has been used in anti-angiogenesis clinical trials for a variety of cancer treatments. Although the early therapeutic effect is ideal, the complicated distant metastasis with time has become the biggest factor limiting efficacy. Li et al. (79) confirm that the knockdown of YAP1 by verteporfin inhibits the proliferation, metastasis and angiogenesis of human umbilical vein endothelial cells (HUVECs), but verteporfin also induces the extracellular release of exosomes with lncRNA MALAT1 highly expressed. Internalized by HCC cells, MALAT1 promotes the metastasis and invasion of tumor cells by activating ERK1/2 signaling. The above mechanism provides a reasonable explanation for the concurrent distant metastasis after verteporfin treatment. Targeted inhibition of the exosomal MALAT1/ERK1/2 signaling axis may improve the long-term survival rate of patients with anti-vascular therapy. EIF3C, the C subunit of the translation initiation complex EIF3, has been confirmed to be abnormally highly expressed in HCC cells, which can stimulate the secretion of tumor cell exosomes, as well as carried into HUVECs and adjacent tissue cells with exosomes. Quantitative proteomics defines S100A11 as a cancer-related protein in HCC exosomes. Studies have shown that the activation of S100A11 expression by eukaryotic translation initiation factor 3 subunit C (EIF3C) promotes the proliferation of HCC cells and the growth of xenograft tumors in nude mice on the one hand. On the other hand, the activation of S100A11 by EIF3C induces tube formation of HUVECs and neovascularization in transplanted matrix gel plugs in nude mice (80). CircRNA-100,338 overexpressed in exosomes derived from highly metastatic HCC cells can activate the metastatic potential of ordinary HCC cells, and has been confirmed to be positively correlated with the metastatic ability of HCC. Mechanistically, exosomal circRNA-100,338 suppresses the formation of vasculogenic mimicry (VM) by negatively regulating Vascular endothelial cell cadherin (VE-cadherin) expression in HUEVCs, and increases the permeability of endothelial cells by destroying tight junctions between HUEVCs. The exogenous silencing of exosomal circRNA-100,338 significantly inhibits the invasion ability of HCC cells, as well as the growth of xenograft tumors in nude mice and the microvessel density in the tissues, meanwhile effectively reducing the number of lung metastatic nodules. In addition, the combined application of exosomal circRNA-100,338 knockdown and IFN-α shows a powerful synergistic effect in reversing exosome-mediated tumor development. Interestingly, further studies have found that the continuous high expression of serum exosomal circRNA-100,338 in HCC patients undergoing radical resection of HCC may be a risk indicator of HCC tissue proliferation, angiogenesis, lung metastasis and poor survival (81). Similar to the above studies, Yan et al. (82) confirm that circ_4911 and circ_4302 are low-expressed in HUVECs. The exogenous upregulation of both can inhibit the tumor formation capacity of HUVECs, increasing the proliferation activity and migration rate, as well as inducing G0/G1 arrest, along with the suppression of epidermal growth factor receptor (EGFR), p38 and cyclin D1. As a ceRNA, hsa_circ_0000092 can mediate the negative regulation of miR-338-3p by competitive combination, which further induces the deinhibition of the downstream target HN1. The oncogene HN1 subsequently participates in the activation of a variety of malignant biological behaviors of HUVECs, including improving the ability of vessel-like tube formation, proliferation, migration and invasion (83). In addition, circGFRA1 can also induce the proliferation and angiogenesis of HCC cells as a molecular sponge by targeted expression inhibition of miR-149 (84). CircASAP1 is clinically closely related to the pulmonary metastasis after radical resection in HCC patients, and is overexpressed in HCC cells with high metastatic potential. Studies have confirmed that it can indirectly induce the overexpression of downstream targets MAPK1 and CSF-1 by acting as the ceRNA of miR-326 and miR-532-5p (both are tumor suppressor factors), thereby regulating miR-326/miR-532-5p/MAPK1 and miR-326/miR-532-5p/CSF-1 signaling pathway respectively to promote the proliferation/invasion of HCC cells and the infiltration of macrophages in the TME. Moreover, the highly expressed circASAP1 exhibits an ideal ability to promote the growth of xenograft tumors *in vivo*, as well as inducing the pulmonary metastasis of HCC cells (85). MiR-451a is abnormally downregulated in HCC pathological tissues and serum exosomes of patients. The co-incubation of HCC-derived exosomes highly expressed miR-451a and HUVECs mediates the transport of miR-451a towards endothelial cell, inhibiting the transfer and tube formation ability of HUVECs and inducing apoptosis. Located on the tight junctions between cells on the surface of the plasma membrane, zonula occludens 1 (ZO-1) serves as an indicator to assess vascular permeability. The overexpressed miR-451a in HUVECs reduces vascular permeability by inducing the transfer of ZO-1 from the cytoplasm to the cell membrane (86). In another study, miR-638 is also significantly downregulated in HCC tissues and serum exosomes. The exogenous upregulation of miR-638 not only antagonizes the proliferation and metastasis of HCC cells through the targeted inhibition of oncogene Sp1 transcription factor (SP1), but also regulates the endothelial function and inhibits angiogenesis *via* the internalization of miR-638 overexpressed exosomes by HUVECs (87).

### Metabolic Reprogramming

Even in the aerobic state, tumor cells still have a tendency to transform from oxidative phosphorylation to glycolytic metabolism. This phenomenon is called the Warburg effect. The metabolic phenotype of aerobic glycolysis can induce tumor cells to form a state of high glucose uptake, providing ATP for tumor proliferation and intermediates/substrates for the synthesis of biological macromolecules (88–91). Meanwhile, pyruvate is converted into lactic acid, which lowers the pH value of the extracellular matrix (92), the acidic tumor microenvironment inducing tumor invasion and metastasis, as well as improving the resistance to ionizing radiation (93, 94). The unique metabolic reprogramming of tumor cells creates a favorable energy supply mode and survival soil for their own growth. Targeted inhibition of the Warburg effect may provide a breakthrough for tumor treatment.

Cytoplasmic Ski and nuclear TRAMP are the two main cofactor complexes involved in the recruitment of target RNA to nuclear exosomes, monitoring and degradation of RNA. RNA helicase MTR4 can mediate RNA insertion into exosomes by binding to TRMAP complex and take part in regulating the stability of exosomal RNA. Research by Yu (95) et al. confirm the mechanism of MTR4 participating in inducing the aerobic glycolysis phenotype of HCC cells: Ectopic expressed in HCC tissues and serum exosomes, MTR4 regulates coordinated alternative splicing by recruiting polypyrimidine tract binding protein 1 (PTBP1) to the intron region of target pre-mRNA. Exogenous knockdown of MTR4 can affect the correct alternative splicing of Glucose transporter 1 (GLUT1)/pyruvate kinase M2 (PKM2) pre-mRNA, thereby reducing the production of functional mRNA, and in turn produce new alternative splicing products GLUT1b and PKM1, both of which severely reduce the glycolysis of HCC. Consequently, the increased oxidative phosphorylation inhibits the rapid proliferation of HCC cells and the growth of xenograft tumors in nude mice. Further studies have found that the oncogenic factor c-Myc can bind to the MTR4 promoter and directly activate the transcription, indicating that MTR4, as a functional mediator of c-Myc, participates in the metabolic reprogramming of cancer cells. Another study confirms the overexpression of circFBLIM1 in the serum exosomes of HCC patients and HCC cell lines, and the co-incubation of isolated serum exosomes with tumor cells can mediate the latter’s internalization of circFBLIM1. As a competitive endogenous RNA, circFBLIM1 deinhibits the expression of the downstream target gene LDL receptor related protein 6 (LRP6) by negatively regulating the expression of miR-338, thereby initiating tumor cell glycolytic metabolism phenotype transformation: High glucose consumption, increased production of lactic acid and ATP, upregulation of ECAR (reflecting the overall glycolytic flux) and downregulation of OCR (reflecting mitochondrial respiration). Additionally, circFBLIM1 also promotes the proliferation of HCC cells and the growth of xenograft tumors, as well as inducing cell apoptosis (96).

Obesity, high body weight, and high-fat diet have all been confirmed to be involved in the occurrence and development of non-alcoholic fatty liver disease (NAFLD), eventually leading to liver fibrosis, cirrhosis and HCC (97, 98). Studies have confirmed that the exogenous stimulation of different pathogenic factors can induce the transformation of healthy adipocytes into cancer-associated adipocytes (CAAs) (99, 100). CAAs can cause local vascular compression through its rapid expansion, forming a tissue hypoxic environment, and participate in inducing fibrosis and abnormal extracellular matrix remodeling, mediating changes in metabolic phenotype, local inflammatory response and neovascularization (101–103). There is a positive correlation between the body fat ratio (BFR) of HCC patients and the level of miR-23 in serum exosomes. Mature adipocytes can achieve targeted transport of miR-23 to HCC cells through exosomal secretion. As a tumor suppressor, von Hippel–Lindau tumor suppressor (VHL) protein participates in mediating the ubiquitination and proteasome degradation of hypoxia inducible factor-1α (HIF-1α), inhibiting the development of HCC. Exosomal miR-23 suppresses the expression of VHL to upregulate HIF-1α, GLUT-1, vascular endothelial growth factor (VEGF) and subsequently triggers the proliferation, metastasis, 5-FU resistance and other malignant biological behaviors of tumor cells (104). The deubiquitinating enzyme ubiquitin specific peptidase 7 (USP7) is overexpressed in a variety of tumor tissues and is closely related to the poor prognosis of patients. Research by Zhang et al. (105) confirms the extremely high expression of circ-DB in serum exosomes and mature adipocytes-derived exosomes of HCC patients with high BFR. The exosomal circ-DB internalized by HCC cells can be used as a molecular sponge to absorb miR-34a endogenously, thereby deinhibiting the downstream target USP7, which inhibits the ubiquitination of cyclin A2 protein to maintain its stability. Both of USP7 and cyclin A2 synergistically promote the proliferation and metastasis of HCC cells, reducing intracellular DNA damage.

### Drug Resistance

Due to the lack of significant clinical symptoms in the early stage of HCC, most patients lose the chance of radical resection. The clinical application of anti-tumor related drugs such as sorafenib, adriamycin (ADM), 5-fluorouracil (5-FU), platinum-containing anti-cancer drugs, camptothecin and gemcitabine has become an important means to extend the survival of patients with advanced HCC (106–108). However, the acquisition of multidrug resistance in tumor cells directly antagonizes the efficacy of the drug and leads to poor prognosis of patients. Existing studies have confirmed that a variety of pathophysiological processes are involved in mediation of tumor cell resistance, including upregulation of drug efflux transporter expression, intracellular agents accumulation and redistribution, inactivation of apoptotic signaling pathways, enhanced DNA damage repair capabilities, acceleration of drug metabolism and cancer stem cells (CSCs) activation (109–111). However, more molecular events related to drug resistance need to be further studied.

The intravenous anesthetic Propofol can inhibit the proliferation of HCC cells by activating the high mobility group AT-hook 2 (HMGA2) and Wnt/β-catenin signaling pathways (112). Co-incubation of the Propofol treated HCC cell-derived exosomes (Propofol-exo) with untreated HCC cells can mediate the inhibition of proliferation, metastasis and invasion of the latter, as well as inducing apoptosis. The exogenous transfection of lncRNA H19 overexpression vector to Propofol-exo can inhibit the expression of miR-520a-3p in HCC cells, so as to activate the expression of the downstream target oncogene LIM domain kinase 1 (LIMK1) and destroy the antagonistic effect of Propofol on the malignant biological behaviors of tumor cells (113).

Zhang et al. (114) and Lou et al. (115) respectively demonstrate the negative correlation between the expression level of miR-199a-3p and cisplatin (DDP)/doxorubicin (Dox) resistance in HCC cells. Internalization of exosomal miR-199a-3p in drug-resistant HCC cells mediates the high drug sensitivity by inhibiting the mTOR pathway, which is manifested as a significant inhibition of proliferation and metastasis after DDP/Dox treatment, accompanied by an increase in apoptotic cell ratio. Meanwhile, exogenous injection of exosomal miR-199a-3p into rats bearing transplantable tumors significantly reduces the size of xenograft tumors and the number of lung metastatic nodules. The expression of miR-744 in Sorafenib drug-resistant cell-derived exosomes is extremely downregulated. Overexpressed miR-744 in drug-resistant cell-derived exosomes can further act as a mediator to induce the upregulation of miR-744 in normal tumor cells, thereby increasing the sensitivity of Sorafenib by inhibiting the protein expression of the downstream target paired box 2 (PAX2) (116). Research by Li et al. (117) reveals the inducing effect of glucose regulates protein 78 (GRP78) on HCC Sorafenib resistance. Through the effective internalization of exosomes in HCC cells, siGRP78 modified exosomes significantly antagonize the Sorafenib resistance of tumor cells. Combination therapy with Sorafenib can inhibit the invasion ability of drug-resistant cells and the degradation of extracellular matrix, reducing the number of liver metastatic nodules in nude mice and inhibiting the liver metastasis of HCC cells. MiR-32-5p is overexpressed in HCC drug-resistant tissues. Exosomes from drug-resistant cells transfer oncogenic miR to sensitive cells and activate the PI3K/Akt pathway by negatively regulating the expression of the downstream target phosphatase and tensin homolog (PTEN), which induces VEGFA-mediated angiogenesis and increases microvessel density in the TME, mediating the multi-drug resistance of HCC (5-FU, OXA, GEM, and Sorafenib) (118).

### Immunologic Escape

The human immune system plays a dual role in tumor progression: On the one hand, it can suppress tumorigenesis through innate and acquired immunity; On the other hand, a large number of immune cells and cytokines recruited in TME mediate the formation of immunosuppressive microenvironment through transformation and modification of a highly plastic system, which leads to immune escape of tumor cells and promotes tumor progression (119). As a negative co-receptor on the surface of activated T cells and B cells, PD-1 induces T cell anergy by binding to its ligand PD-L1, thereby maintaining the balance between autoimmunity and immune tolerance (120–122). Tumor cells inhibit the cytotoxicity of CD8+ T cells through the highly expressed PD-L1 to mediate immune escape.

As a highly plastic functional heterogeneous cell group, macrophages play an important role in the construction of TME. Exogenous stimulation of the microenvironment mediates the polarization of macrophages into two different phenotypes: M1 macrophages trigger a rapid pro-inflammatory response and eliminate pathogens, exhibiting anti-tumor activity, while M2 macrophages promote tumor development due to the activities of anti-inflammation and tissue repair. The polarization from M1 to M2 phenotype has become a driving factor for cancer development. HCC cells deliver ectopic expressed lnc TUC339 to nearby macrophages through exosomes, and participate in the repolarization of macrophages from M1 to M2. The exogenous silencing of lnc TUC339 by siRNA not only induces the upregulation of the pro-inflammatory cytokines IL-1β, TNF-α and the costimulatory molecule CD86 in macrophages, realizing the reactivation of macrophages, but also induces macrophages from M2 repolarizes to M1, thereby activating its cytotoxic effect on tumor cells (123). The abnormal high-expression of related markers GRP78, ATF6, PERK, IRE1α confirms the occurrence of endoplasmic reticulum stress events in HCC. The upregulation of ER-related proteins is not only closely related to the poor overall survival and pathological scores of HCC patients, but also indicates the high infiltration of CD68+ macrophages in the TME. The internalization of HCC-derived exosomal miR-23a-3p by macrophages mediates the inhibition of PTEN expression, while upregulating AKT phosphorylation and PD-L1 expression subsequently. Further studies have confirmed that the co-culture of macrophages activated by exosomes with T cells downregulates the ratio of CD8+ T cells and inhibits the production of interleukin 2, as well as inducing T cell apoptosis (124).

**Figure 2:**
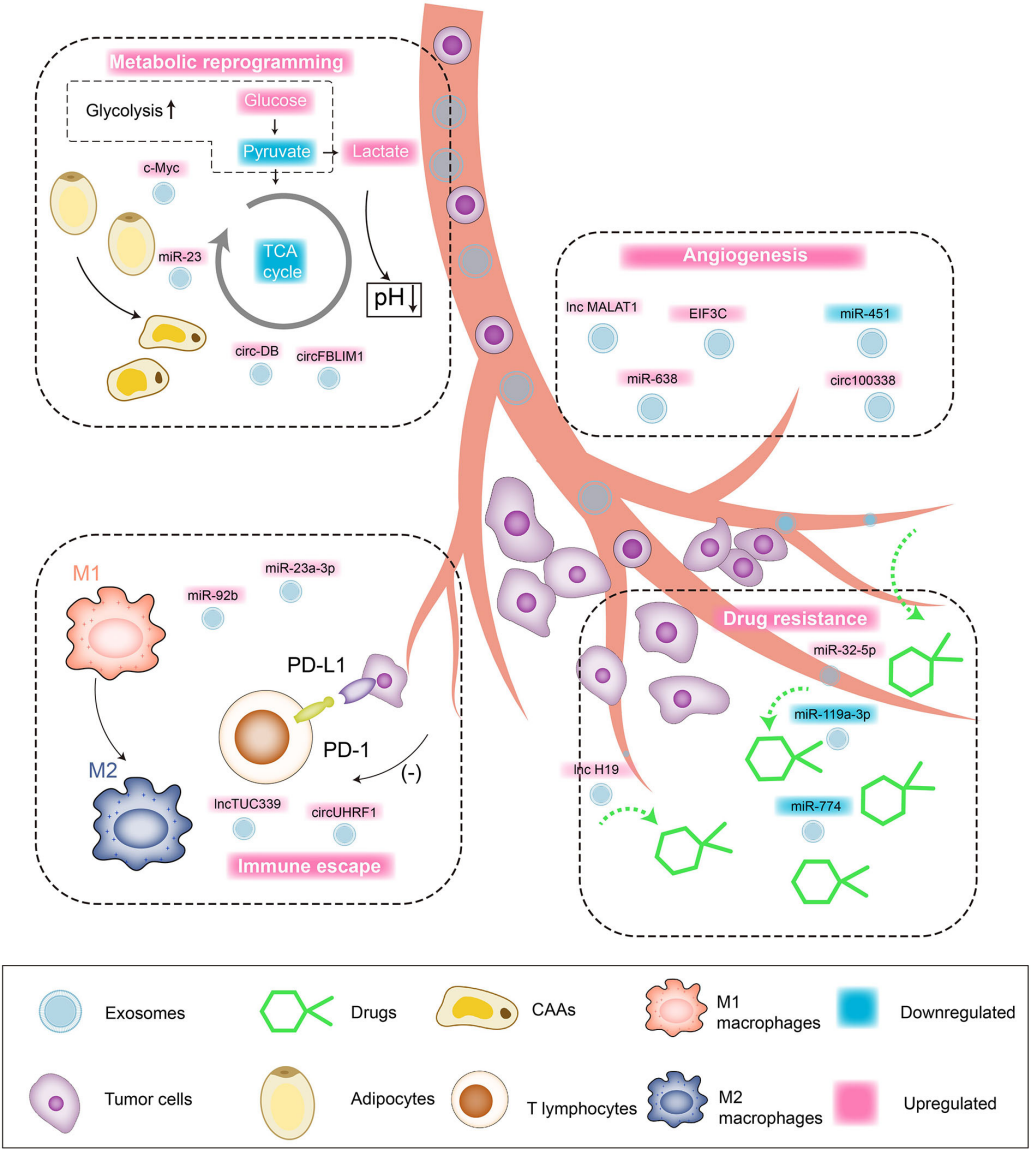
Interaction network between exosomal ncRNAs and tumor microenvironment of HCC. Tumor cell/non-tumor cell-derived exosomes regulate the malignant biological behaviors of HCC through targeted cargo transportation. 1) Angiogenesis: upregulated lnc MALAT1, miR-638, EIF3C, circ100338 promote the angiogenesis in tumor microenvironment and HCC cell proliferation/blood-metastasis. 2) Drug resistance: upregulated miR-32-5p and lnc H19 are involved in mediating the drug resistance of HCC cells. 3) Metabolic reprogramming: upregulated c-Myc and circFBLIM1 induce aerobic glycolysis (Warburg Effect) of HCC cells. Upregulated miR-23 and circ-DB promote the transformation of fat cells into tumor-associated tumor cells (CAAs). 4) Immune escape: upregulated lnc TUC339 induces the repolarization of macrophages from M1 to M2, and the upregulation of miR-23a-3p can promote PD-L1/PD1 binding and induce T cell apoptosis.

As one of the main inhibitory receptors of NK cells, TIM-3 realizes the imbalance of anti-tumor immunity of NK cells by binding to tumor cells or ligands in the microenvironment. Carried by exosomes to NK cells, the overexpressed circUHRF1 achieves the same trend expression with TIM-3 by competitively binding miR-449c-5p, mediating the downregulation of active NK cell ratio in the microenvironment and the low secretion of IFN-γ and TNF-α through the induction of TIM-3. In addition, clinical pathological studies indicate that the expression level of serum circUHRF1 is significantly negatively correlated with the number of NKG2D-positive cells. Patients with overexpressed circUHRF1 have shown obvious PD1 resistance and poor clinical prognosis (125). HCC patients after living donor liver transplantation (LDLT) still have a high risk of recurrence (3–18%), while the underlying predisposing factors are still unknown yet. Nakano (126) et al. separately extract serum and exosome-free serum from orthotopic liver cancer rat models and inject them into nude mice intravenously. The former serum shows high levels of AFP and overexpressed miR-92b in tumorigenic tissues, while the latter shows no abnormalities of ATP levels. ROC curve confirms that the serum miR-92b level of patients after ADLT has an ideal predictability for the early (AUC = 0.925) and late (AUC = 0.761) recurrence of HCC. Mechanically, serum CD69 is not only involved in lymphocyte proliferation induction and signal transduction as one of the surface antigens of activated T cells, but also participates in NK cell-mediated cytotoxicity as an important mediator. The overexpressed miR-92b in HCC-derived exosomes actively infiltrates NK cells in the tumor microenvironment, participating in the inhibition of CD69 expression and antagonizing the cytotoxic effects of NK cells on HCC, thereby inducing immune escape of tumor cells. In summary, the regulatory effect of exosomal ncRNAs on HCC is shown in **Figure 2**.

## Exosomal NcRNAS as Early Diagnostic Biomarkers

Ultrasound, as the preferred test for surveillance of HCC, limited by operator dependency and diagnostic accuracy, exhibits unsatisfactory clinical results (127). Alpha-fetoprotein (AFP), the most commonly used serological test, failed to show good diagnostic efficacy in retrospective case-control studies (Sensitivities: 60%, Specificities: 80%) (128, 129). Although the combined use of ultrasound and AFP can increase the detection rate, it may also increase false-positive suspicions and cost (130). Other tumor biomarkers, such as des-γ carboxyprothrombin, lectin-bound AFP, glypican 3, Golgi protein 73, and Dickkopf 1, also fail to effectively improve the accuracy of diagnosis (128, 129, 131, 132). Therefore, there is an urgent need for reliable inspection methods for early HCC diagnosis and tumor recurrence monitoring. The liquid biopsy technology applied to the analysis of human non-solid biological tissue samples (such as blood, cancerous ascites, feces, etc.) has received widespread attention in recent years (133). The collection of tumor genetic material in body fluids overcomes the invasive inconvenience of conventional biopsy and the limitations without being able to collect multiple times, providing an opportunity to systematically and dynamically tracking the evolution of the genome (134). As one of the cornerstones of liquid biopsy, exosomes have shown good diagnostic performance in growing HCC-related studies, which are expected to provide more detailed and personalized decisions for cancer management.

Cho et al. (135) comprehensively analyze the miR expression profiles of three different human liver cancer RNA sequencing datasets, and use logistic regression models to develop serum exo-miR panels. Four serum exo-miRs (miR-25-3p, miR-1269a, miR-4661-5p and miR-4746-5p) performed ideally in the test cohort (AUC >0.8). Noteworthily, serum exo-miR-4661-5p in all stages of HCC (AUC = 0.917), even early stage (AUC = 0.923), shows good diagnostic performance, whose accuracy is higher than other candidate serum exo-miRs and serum AFP. Furthermore, the exo-miR panel composed of exo-miR-4661-5p and exo-miR-4746-5p is considered to be the most accurate biomarker of early HCC (AUC = 0.947). The S fragment of RN7SL1 is enriched in HCC exosomes, with significantly higher expression level than that in the healthy donor group. ROC curve confirms that compared to the full-length transcript of RN7SL1 (AUC = 0.75), the S fragment of serum RN7SL1 is a better diagnostic marker for HCC (AUC = 0.87) (136). Ghosh et al. (137) validate that the expression level of miR-21-5p/miR-10b-5p/miR-221-3p/miR-223-3p in serum exosomes of HCC patients are significantly higher than that in patients with chronic hepatitis. The combination of miR-21-5p+miR-10b-5p+miR-221-3p+miR-223-3p can be used to distinguish HCC from chronic hepatitis and chronic hepatitis + liver cirrhosis (AUC = 0.86). Meanwhile, miR-10b-5p+miR-221-3p+miR-223-3p shows better efficacy in distinguishing low AFP-HCC from non-HCC hepatitis or liver cirrhosis patients compared to the combination of the four miRNAs (AUC = 0.84). Eight exosomal miRNAs (miR-122, miR-125b, miR-145, miR-192, miR-194, miR-29a, miR-17-5p and miR-106a) are overexpressed in the serum of HCC patients, ROC curve analysis confirms that the above exosomal miRNAs are potential biomarkers for distinguishing HCC patients from healthy controls (AUC = 0.535–0.850) (138). The expression of exosomal miR-212 in the serum of patients with chronic hepatitis B, liver cirrhosis and stage II/III HBV-HCC/Non-HBV-HCC are all significantly upregulated. Compared with AFP and CA125, miR-212 has a more significant correlation with Child-Pugh Classification, MELD score and MELD score, as well as associated with the progression of hepatitis B-related liver cancer, esophageal varices, ascites, and liver cirrhosis progress associated with HBV infection. The diagnostic value of miR-212 for HBV-HCC (AUC = 0.886) is higher than that of AFP (AUC = 0.849) and CA125 (AUC = 0.780), but for Non-HBV-HCC. The diagnostic value of miR-212 (AUC = 0.793) is slightly lower than AFP (AUC = 0.849) and higher than CA125 (AUC = 0.777) (139). Research by Huang et al. (140) reveals that serum exosomal lnc85 is highly expressed in both AFP-positive and negative HCC patients, and can distinguish AFP-negative HCC patients from healthy controls and liver cirrhosis patients (AUC = 0.869). The expression levels of miR-122, miR-148a and miR-1246 in HCC serum exosomes are higher than those in the liver cirrhosis and normal control groups, but fail to show significant differences compared with the chronic hepatitis group. MiR-148a shows good efficacy in identifying early HCC and liver cirrhosis (AUC = 0.891), which is significantly better than AFP (AUC = 0.712), and the combination of miR-122, miR-148a and AFP can further increase AUC to 0.931. Additionally, miR-122 is ideal for distinguishing HCC and NC (AUC = 0.990) (141). The multiple serum exosomal ncRNAs will undoubtedly open up a broad prospect for the clinical application of exosome-based liquid biopsy technology in the early diagnosis of HCC.

## Therapeutic Functions of Exosomal NcRNAS on HCC

Due to the lack of obvious clinical symptoms in the early stage and the high diagnosis rate at advanced stage, surgical resection and liver transplantation have become the main treatment methods for HCC (142). NcRNAs can shuttle between tissue cells with exosomes, regulating the biological behavior of recipient cells through intercellular communication. Abounding ncRNAs can be used as tumor suppressor regulators to participate in the rate-limiting step of tumor development. The targeted delivery of therapeutic ncRNAs carried by exosomes to tumor cells mediates its growth inhibition and achieves personalized treatment of different tumor cells (143).

As one of the key sources of adult stem cells, mesenchymal stem cells (MSCs) have become the ideal seed cells for tissue engineering due to self-renewal, multi-differentiation potential and low immunogenicity (144, 145). In addition, the high secretion properties of exosomes make MSCs a good place to cultivate therapeutic exosomal ncRNAs (146). MSCs-derived exosomes pass through the blood tissue barrier due to the small size and are easily absorbed by tissue cells (147). They induce HCC cell apoptosis and cell cycle arrest *in vitro* and *in vivo* respectively (148), increasing the TME/systemic circulating NK cell ratio (149) to limit tumor progression. Cancer stem cells (CSCs) may be derived from the malignant transformation of tissue colonized stem cells (NSCs) (150). Exosomes secreted by CSCs are important mediators of chemotherapy resistance and tumor metastasis. The lncRNA H19 derived from CD90+CSC exosomes can induce angiogenesis, which limits the efficacy of anti-angiogenic therapy in HCC (151). In addition, CSCs-derived exosomes effectively increase the levels of serum tumor markers (AFP and GGT) and liver enzymes (ALT, AST and ALP) in DEN-induced HCC rats, as well as increasing the number of metastatic of cancer nodules. Meanwhile, CSCs-derived exosomes also significantly inhibit apoptosis, inducing angiogenesis, metastasis, invasion and EMT of HCC cells. MiR-21, lnc Tuc339, lnc HEIH and lnc HOTAIR are extremely upregulated in HCC exosomes affected by CSCs exosomes, while miR122, miR148a, miR16, and miR125b exhibit significant downregulation effect. Interestingly, MSC-derived exosomes reverse the above-mentioned effects and exhibit significant anti-tumor ability (152). The downregulation of miR-451a in HCC tissues indicates poor prognosis, and is closely related to malignant biological behaviors such as larger tumor diameter, no tumor capsule, advanced TNM stage, high differentiation and portal vein injury. Being transfected with an overexpression vector, miR-451a highly expressed in MSCs can be carried to HCC cells along with exosomes, inhibiting the paclitaxel resistance, proliferation, metastasis and EMT of tumor cells by negatively regulating the expression of ADAM metallopeptidase domain 10 (ADAM10) (153).

Oncogenic ncRNAs carried by tumor cell-derived exosomes (TDEs) can mediate their malignant biological behaviors through the internalization of different receptor cells. The suppression of TDEs dissemination from the primary tumor has become an important challenge in the treatment of cancer (154–156). Although therapeutic agents that block RAB family proteins, SNARE family proteins or p53 can reduce the secretion of TDEs (157, 158), the inhibitory effect on the secretion of exosomes in non-target tissues can also impair normal physiological functions (159). Liang et al. (160) prepare DC/CS-NP nanoparticles that can effectively encapsulate siRNA by thin-film hydration, which can rely on their own lipid bilayer structure to avoid siRNA degradation in the circulating or endosomal environment and ensure siRNA release at the targeted location. As a representative oncogenic exosomal miRNA, miRNA-21 is significantly upregulated in HCC cells. DC/CS NPs carrying Sphk2 siRNA induces miRNA-21 assembly to decrease in exosomes by inhibiting the expression of the cargo molecule packaging protein S1P, thereby destroying the carcinogenic efficacy of TDEs and avoiding the canceration of normal cells. Osteopontin (OPN), an important cell cancer assessment marker, shows no upregulation in the DC/CS-siSphk2 NPs treatment group, demonstrating the satisfying targeted therapeutic ability of nanoparticle.

The aerobic glycolysis of cancer cells (Warburg Effect) induces the acidification of the TME (161). Combined with abnormal blood perfusion and local hypoxia of tumors, a pH gradient is generated from the area inside the tumor to the normal tissues adjacent to the cancer, creating an ideal breeding ground for the metastasis of tumor cells (162, 163). Acidic conditions induce the upregulation of HIF-1α and HIF-2α in HCC cells, which bind to the miR-21 and miR-10b promoter regions to activate the transcription. The overexpressed miR-21 and miR-10b in HCC-derived exosomes induce metastasis, invasion and EMT of adjacent cells in a paracrine manner. Tian et al. (164) develop a nano-drug targeting exosomal miR-21 and miR-10b based on the PDCM system, and demonstrate obvious tumor growth inhibitory effects *in vivo*, effectively reducing the number of lung metastatic nodules. The low pH environment is conducive to the release of exosomes and the uptake of receptor cells (165). The neoplastic vacuolar H+ATPase (V-ATPases) overexpressed in cancer cells is very analogous with the H+/K+ATPase in parietal cells, which can rely on ATP to achieve H+ transport (166). The proton pump inhibitor pantoprazole effectively improves the liver function (ALT, albumin, T bilirubin, D bilirubin and AFP) in rats with precancerous lesions and the microstructure characteristics of the liver, reducing the exosomal abundance in the HCC tissues and serum of the rat liver. Meanwhile, pantoprazole also downregulates the serum exosomal RAB11A mRNA and lncRNA RP11-513I15.6, while upregulating exosomal miRNA-1262 in a dose-dependent manner (167).

Radiofrequency ablation (RFA) is one of the ideal treatments for advanced liver cancer besides surgical resection. However, many studies suggest that it is difficult for patients with liver cancer to achieve adequate ablation (168). Insufficient ablation area (169), heat loss of the tumor through blood vessels (170), or low temperature area in large tumors during ablation (171) may all be the inducing factors for insufficient ablation. The high recurrence and metastasis of residual tumors after RFA deficiency have always been a research hotspot in the academic field. Ma et al. (172) use heat-treated HCC cells to simulate the RFA environment. Studies confirm that heat-treated cells-derived exosomes mediate the upregulation of MYC expression in normal HCC cells, which bind to the ASMTL-AS1 promoter and positively activate its transcription. ASMTL-AS1 plays a role of carcinogen: As the disease stage progresses, ASMTL-AS1 in serum exosomes of HCC patients shows an increasing trend with further increasing after RFA; clinicopathological studies have confirmed that the expression level of ASMTL-AS1 is closely related to tumor volume, distant metastasis, and TNM staging. Meanwhile patients with highly expressed ASMTL-AS1 have significantly poor clinical prognosis. In terms of mechanism, as an endogenous molecular sponge, ASMTL-AS1 targets miR-342-3p and upregulates the expression level of downstream target nemo like kinase (NLK). Highly expressed NLK activates the malignant biological phenotypes of HCC cells by mediating Ser128 phosphorylation of Yes1 associated transcriptional regulator (YAP) and nuclear translocation, which are manifested in the promotion of proliferation, metastasis, invasion and the occurrence of EMT. The above studies reveal that the MYC/ASMTL-AS1/miR-342-3p/NLK/YAP regulatory axis plays a crucial role in mediating the malignant biological behaviors of tumor cells after RFA deficiency, which indicates that the combination of targeted therapy against molecular targets can provide support for the prognosis of patients after RFA. The above and other therapeutic ncRNAs/drugs and related targets are summarized in **Table 2**.

**Table 2:** Therapeutic target of exosomal ncRNAs for HCC.

Therapeutic substances	Functions	Targets/Substrates	References
**ncRNAs**			
miR-451a	Inhibiting paclitaxel resistance, proliferation, metastasis and EMT of tumor cells, as well as inducing apoptosis and cell cycle arrest	ADAM10	(153)
miR-145	Inhibiting the proliferation and metastasis of HCC cells, antagonizing the growth rate of xenograft tumors and the number of lung metastases in nude mice	GOLM1/GSK-3b/MMPs	(47)
miR-125	Inhibiting the proliferation and sphere formation of HCC cells	CD90	(50)
lncRNA H19	Inducing HCC cell apoptosis, inhibiting angiogenesis, invasion, metastasis and EMT, as well as reducing serum markers (AFP and GGT) and liver enzyme levels (AFP and GGT) in HCC rats	miR-21, lnc Tuc339, lnc HEIH, lnc HOTAIRmiR122, miR148a, miR16, miR125b	(151)
SENP3-EIF4A1	Inhibiting the proliferation and metastasis of tumor cells, and reducing the volume and weight of xenograft tumors in nude mice	miR-9-5p/ZFP36	(36)
circ-0051443	Inhibiting the proliferation of HCC cells, mediating G0/G1 cell cycle arrest and apoptosis, as well as reducing the weight and volume of xenograft tumors in nude mice	miR-331-3p/BAK1	(57)
**Drugs**			
nano-drugbased on the PDCM system	Inhibiting the growth rate of xenograft tumors, reducing the number of lung metastatic nodules, and improving the acidic environment of tumor microenvironment through targeted inhibition of exosomal miR-21 and miR-10b secreted by HCC cells	Exosomal miR-21, miR-10b	(164)
Pantoprazole	Improving liver functions (ALT, albumin, T bilirubin, D bilirubin, and Alfa Fetoprotein AFP) and liver microstructure characteristics of precancerous lesion rats, and improving the acidic tumor microenvironment.	Exosomal RAB11A mRNA, lncRNA RP11-513I15.6, miRNA-1262	(167)
Sphk2 RNAi nanoparticles	Inducing a reduced packaging of miRNA-21 in exosomes by inhibiting the expression of packaging protein S1P, subsequently inhibiting the carcinogenic efficacy of TDEs and avoiding normal cell canceration.	Exosomal miRNA-21	(160)

## Conclusion and Perspectives

As small EVs, multi-cell-derived exosomes build a communication bridge between tissue cells. Intercellular signal transduction through cargo transportation induces the physiological or pathophysiological processes of different recipient cells. NcRNAs, the most abundant biologically active substances in exosomes, are involved in regulating the complex interaction between HCC cells and TME, as well as mediating HCC development through certain specific methods (tumor angiogenesis, metabolic reprogramming, drug resistance and immune escape). Relevant research on its specific regulatory mechanism can provide potential targets for precision treatment of HCC. Meanwhile, liquid biopsy technology based on exosomal ncRNAs also provides a non-invasive and promising option for early diagnosis and prognostic evaluation of multiple malignant tumors. What is more noteworthy is that due to its unique advantages, exosomes have developed into natural vehicles for the targeted delivery of therapeutic biologically active substances, bringing the gospel for precise treatment and effective survival of patients with advanced HCC. Its advantages are as follows: 1) As natural carriers produced by endogenous cells, exosomes have high stability and low immunogenicity, which can establish good biocompatibility with the body’s immune system, and have low toxic/side effects. 2) Exosomes can avoid the phagocytosis of macrophages, penetrating blood vessels to enter the extracellular matrix, exhibiting efficient substance delivery capabilities. 3) Exosomes can break through biological barriers such as the blood-brain barrier to treat a variety of refractory diseases. However, although exosomes exhibit unique advantages in the delivery of numerous substances, their potential safety issues and industrialization issues are still stumbling blocks hindering their clinical application: 1) There is still a lack of standardized exosome separation technology. In view of the variability of biological fluids and exosome abundance, it is necessary to adopt standardized exosome isolation methods to ensure a consistent and reproducible supply of exosomes. 2) The development of exosomal biomarkers such as RNAs lacks large-scale prospective studies to provide evidences that liquid biopsy can replace tumor tissue biopsy. 3) Exosomal preparations should be based on the premise of high quality, uniform properties and no other cell vesicle contamination, and a complete quality control system needs to be established to improve the safety of their biological applications. However, in order to meet the above challenges, researchers have developed some engineering methods for magnetic separation or biotin separation to simplify the separation steps involved in the preparation of exosomes. Meanwhile, with the progress of nanomedicine and material manufacturing methods, many technologies will be used to design exosomes according to the specific characteristics of individual diseases to realize their large-scale production and rapid separation. Engineered exosomes may be developed as smart drugs for clinical diagnosis and treatment in the near future. In summary, with the continuous advancement of basic research on exosomes and the continuous development of modern biotechnology, engineered exosomes will have broad application prospects in the field of tumor treatment and regenerative medicine in the future.

## Author Contributions

HX wrote the manuscript. YX, JY and YC revised and approved the manuscript. All authors contributed to the article and approved the submitted version.

## Funding

This study was funded by Natural Science Foundation of China (81902431), China Postdoctoral Science Foundation (2019T120279, 2018M641849), Foundation of Key Laboratory of Myocardial Ischemia, Ministry of Education (KF201810), Heilongjiang Postdoctoral Science Foundation (LBH-Z18107), Outstanding Youth Project of Natural Science Foundation of Heilongjiang (YQ2019H007).

## Conflict of Interest

The authors declare that the research was conducted in the absence of any commercial or financial relationships that could be construed as a potential conflict of interest.
